# Quantitative characterization of non-DLVO factors in the aggregation of black soil colloids

**DOI:** 10.1038/s41598-022-09067-2

**Published:** 2022-03-24

**Authors:** Xiaodan Gao, Qi Kou, Kailu Ren, Yan Zuo, Yingde Xu, Yun Zhang, Rattan Lal, Jingkuan Wang

**Affiliations:** 1 Northeast Key Laboratory of Conservation and Improvement of Cultivated Land (Shenyang), Ministry of Agriculture, College of Land and Environment, Shenyang Agricultural University, Shenyang, 110866 China; 2 Carbon Management and Sequestration Center, The Ohio State University, Columbus, OH 43210 USA

**Keywords:** Environmental sciences, Solid Earth sciences

## Abstract

The variable role and fate of soil colloids under different environmental conditions are derived from their dispersion and aggregation properties. In this work, dynamic and static light scattering were used to characterize the original size, aggregation kinetics of natural black soil colloids (BSCs) and structural features of aggregates in electrolytes with different cations (K^+^, Mg^2+^, Ca^2+^), respectively. For these three cations, the aggregation kinetics followed the trend of Ca^2+^ > Mg^2+^ > K^+^ and the critical coagulation concentration (CCC) followed the sequence: K^+^ (134.30 mmol L^−1^) > Mg^2+^ (13.27 mmol L^−1^) > Ca^2+^ (4.19 mmol L^−1^). The results indicated that the aggregation behavior in different valence cation systems followed the classical Derjaguin-Landau-Verwey-Overbeek (DLVO) model qualitatively. However, the quantitative differences of CCC suggest the existence of ion-specific effects. The effective ionic charge coefficient 1.31, 2.20, and 2.78 of K^+^, Mg^2+^ and Ca^2+^ were proposed to consider of all the non-DLVO factors, which were obtained by forming a relationship based on mathematic between the electrostatic repulsion and the van der Waals attractive interaction at the CCC. The non-classical polarization of cations in a strong soil electric field is a primary mechanism of cation effects on soil colloid interactions, causing the difference in colloid interaction energy and further affecting soil colloid aggregation. This result is crucial for enriching the theory of charged colloidal interactions.

## Introduction

Natural soil colloids, with a size ranging from 1 to 1000 nm, are the most active components in soil, and play fundamental roles in soil fertility and environmental governance^1–5^. Under natural conditions, colloids can be released from the soil solid matrix and interact with the surrounding environments^6^. Colloidal particles in aquatic environment are the carriers of heavy metal ions and nutrient elements, leading to colloid-driven transportation when the colloids are stable^2,4,7^. Depending on the stability of soil aggregates, the dispersion and mobilization of soil colloids are also related to soil erosion^8–11^. Therefore, the stability or aggregation of soil colloids has caused several environmental concerns.

The classical Derjaguin-Landau-Verwey-Overbeek (DLVO) model is widely used to simulate the aggregation behavior of soil colloids in different electrolytes^12–14^. In general, divalent cations (Mg^2+^ and Ca^2+^) destabilize soil colloid particles more efficiently than monovalent cations (K^+^ and Na^+^) due to a more intensely compressed electrical double layer (EDL), the Schulze-Hardy rule and DLVO theory can be used to explain it^15–18^. However, divalent ions have different surface affinities, leading to a different aggregation process^19^. It has been reported that the DLVO model fail to describe the colloid behavior in aqueous media fully^20^. Chorom and Rengasamy^21^ concluded from a mineral colloid dispersion that the net resultant force derived from the DLVO forces dominates the soil colloidal dispersion and aggregation, but the net resultant force is affected by non-DLVO factors. In environmental systems, there are many interactions (e.g., hydrogen bonding and the hydrophobic effect, hydration pressure, non-charge transfer Lewis acid base interactions, and steric interactions). However, the traditional DLVO model do not include these interactions, which also play an essential role^20,22^. Among them, the theory that takes short-range Lewis acid–base interactions into account is defined as extended DLVO (EDLVO) theory^23,24^. Nevertheless, several studies have also shown that critical coagulation concentration (CCC) even in the same valence ions inducing the aggregation of nanoparticles can be able to show different^25,26^, which is referred to as the specific ion effect or the Hofmeister effect. The current theories state that for a charged colloidal particle system, strong cationic polarization in cation–surface interactions strongly influences the surface properties of the colloid and the interaction energy between particles and thus, govern the stability of the colloid suspension^11,27^. The strong polarization increases with the increase in electric field strength or decreases with the external cation concentration^27^. Thus, polarization of ions at the interface may also be considered as one of the non-DLVO factors.

In general, it is customary to select one or two separate mineral or organic soil components as model materials. However, the mature soil is poly functional and contains compounds with diverse composition and structure. Therefore, it is appropriate to conduct research on the aggregation behavior using real soil particles. The black soil in northeast China mostly has both permanently and variable charge on their surface because of a high content of mineral and organic components. The presence of natural organic matter, with a soft multibranched structure and high molecular weight and surface charge number, can both significantly inhibit the aggregation potential of mineral colloids/nanoparticles^28,29^, and also act as a binding substance promoting the formation of soil aggregates in natural soils^30,31^. The electric field strength at the surface of natural soil particles can reach 4 × 10^8–9^ V m^−1^.^32,33^. Some pertinent research questions which need to be addressed include the followings: What’s the aggregation response of the black soil particles with higher organic content in strong soil electric field? How strong the non-DLVO factors affect soil colloid dispersion and soil aggregate structure?

The purposes of this study were to: (1) clarify the specific ion effects of K^+^, Mg^2+^ or Ca^2+^ on natural soil colloid aggregate stability; (2) quantitatively characterize the effect of non-DLVO factors on particle interaction while considering the specific ion effects and try to explore their main sources. The results can strengthen the understanding of the effects of ion species on the colloid aggregation process.

## Materials and methods

### Preparation and characterization of black soil colloids

The soil colloids were extracted from the black soil (Luvic Phaeozem, FAO soil classification) in Harbin (45° 19′ N, 126° 18′ E), Heilongjiang Province, Northeast of China. The black soil developed from a loess-like parent material and Maize (*Zea mays* L.) is the principal crop of this region. The soil sample from the top 0–20 cm layer was collected and sieved through a 0.25 mm mesh after air dried. Then the black soil colloids (BSCs) were prepared following the procedures^14,34^: first, 50.0 g black soil was transferred into a 500-mL beaker containing 500 cm^3^ of deionized water. After thoroughly stirring the mixture, the pH of the suspension was adjusted to 7.5 ± 0.1 by 0.5 mol L^−1^ KOH solution. Second, the soil suspension was intensively sonicated (Scientz-IID, Ningbo, China) for 15 min and then diluted to 5000 mL with ultrapure water. Third, the < 200 nm (hydrodynamic diameter) fraction was extracted following the static sedimentation method^14^, and electrodialysis was used to remove free ions from the solution. A particle concentration of ~ 0.8185 g L^−1^ was determined by the oven-dry method. The specific area and CEC of BSCs determined by combined measurements are 180 m^2^ g^−1^ and 24.2 cmol_(+)_ kg^−1^, respectively^35^. Which are now ready for dynamic light scattering (DLS) experiments. Moreover, < 2 μm soil colloid suspension was extracted also for measurement of the zeta potential.

### Dynamic and static light scattering measurements

**Figure 1:**
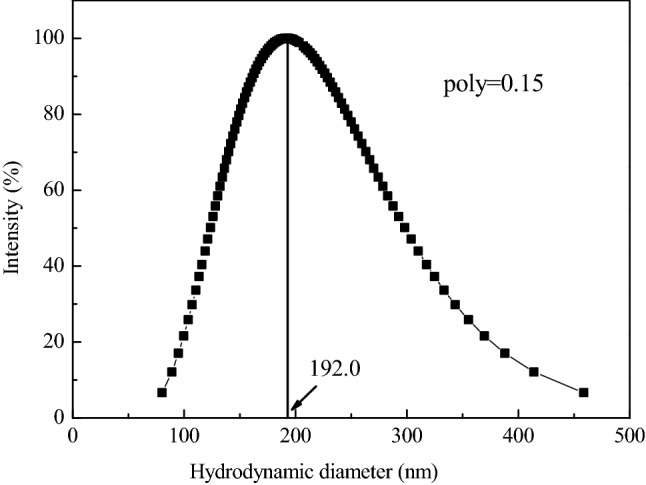
The size distribution of the primary black soil colloid particles.

DLS was used to measure the hydrodynamic diameter of particles. A BI-200SM multiangle laser light scattering instrument (Brookhaven, USA) was used for the DLS measurements. All the measurements were carried out at wavelength of 532 nm and scattering angle of 90°. The scattering instrument was switched on to warm up for at least 25 min before an experiment. A total of 2 cm^3^ of the BSC suspension was add into the scattering bottle. In order to attain the electrolyte concentration that experiment required and a total volume of 10 mL, moderate electrolyte solution and deionized water should be added to the bottle too. The BSC particle concentrations in the suspension were 0.1637 g L^−1^. The average hydrodynamic diameter of the original BSCs measured by DLS was 192.0 nm (Fig. 1). Time-resolved DLS was used to measure the change in the number-weighted average hydrodynamic diameter of BSCs and their aggregates as a function of time in the presence of different cations. The size of the BSC aggregate was recorded every 30 s for 60 min. All measurements were carried out at 25 ℃.

Electrolyte solutions (KCl, MgCl_2_ or CaCl_2_) were passed through a 0.22 μm microfiltration membrane before use. The electrolyte concentrations gradient at the pre-experiment stage ranged 30, 50, 60, 80, 100, 150, 200, 300 mmol L^−1^ for KCl; 1, 2, 3, 4, 5, 8, 10, 20 mmol L^−1^ for MgCl_2_; and 0.5, 1, 1.5, 2, 3, 4, 5, 10 mmol L^−1^ for CaCl_2_.

### Determinations of the critical coagulation concentration (CCC) and the activation energy of aggregation

The stability of the colloidal suspension in the electrolyte solution is usually characterized by CCC^36^. Researchers established a method for determining the CCC of a polydisperse nonspherical colloid suspension by DLS measurements^36^. Then, the activation energy of particle aggregation was further measured^27^. In this study, we measured the total average aggregation rates (TAA) at different electrolyte concentrations, and then estimated the CCC. The TAA rate was defined as per Eq. (1):

1 $$\tilde{v}_{T} (f_{0} ) = \frac{1}{{t_{0} }}\int\limits_{0}^{{t_{0} }} {\tilde{v}(t,f_{0} )\;dt} = \frac{1}{{t_{0} }}\int\limits_{0}^{{t_{0} }} {\frac{{D(t) - D_{0} }}{t}\;dt}$$

where,  *ṽ*_*T*_(*f*_0_) (nm min^−1^) is the rate of change of TAA in the time range from* t* = 0 to *t* = *t*_0_, *f*_0_ (mmol L^−1^) is the electrolyte concentration, *D*(*t*) (nm) and *D*_0_ (nm) represent the aggregate hydrodynamic diameter at *t* and *t*_*0*_, respectively. Both of them can be directly obtained by DLS, and the *t*_0_ as integral upper limit in Eq. (1) in the present study was 60 min.

The activation energy of aggregation in suspension was estimated using Eq. (2):

2 $$\Delta E(f_{0} ) = - kT\ln \frac{{\tilde{v}_{T} (f_{0} )}}{{\tilde{v}_{T} (CCC)}}$$

where, Δ*E*(*f*_0_) (J mol^−1^) is the activation energy, *T* (K) is the absolute temperature, *k* (J K^−1^) is the Boltzmann constant, and  *ṽ*_*T*_(*CCC*) is the TAA rate at *f*_0_ = CCC.

### Electron microscopy and static light scattering characterize

The surface morphology and elemental composition of aggregates were carried out using field-emission scanning electron microscopy (FESEM) (Zeiss Ultra Plus). Specimens were sprayed with gold in a vacuum coating machine for 120 s, and examined at 2–30 kV. The analysis showed that the semi-quantitative elemental contents of BSCs were 24.74% of Si and 39.33% of O, followed by 13.26% of Al, 8.98% of Fe, 7.17% of C, 1.06% of Ca, 0.73% of N, and 0.23% of Mn. Predominant clay minerals were illite and montmorillonite.

The static light scattering (SLS) measurement was used to characterize the structure of aggregates formed under different electrolyte conditions. The scattering index was obtained by measuring the change of the scattering light intensity with the scattering vector in the BSC aggregation process. When the scattering index is a constant, it is the fractal dimension (*d*_*f*_) of the aggregate. The *d*_*f*_ was used to characterize the structural compactness of the formed aggregates.

### Zeta potential measurements

The zeta potential of soil particles was measured by a Zeta Plus (Brookhaven, USA). Negatively charged surfaces were observed for the black soil colloid with a zeta potential value of − 26.34 mV at pH = 7. The suspensions were prepared by adding 1 cm^3^ of the ˂ 2 μm BSC suspension (1.255 g L^−1^) into 9 cm^3^ of different species and concentration electrolyte solutions, namely, 30–350 mmol L^−1^ KCl and 0–10 mmol L^−1^ CaCl_2_ and MgCl_2_ under neutral pH conditions. Each measurement was performed 3–10 times at room temperature (25 ℃).

### Quantitative calculation DLVO forces

The DLVO theory treats the interaction between particles is according to the balance between van der Waals attraction, *P*_*vdW*_(*λ*) (atm), and electrical double-layer repulsion, *P*_*EDL*_(*λ*) (atm); expressed as per Eq. (3):

3 $$P_{DLVO} (\lambda ) = P_{vdW} (\lambda ) + P_{EDL} (\lambda )$$

where *P*_*DLVO*_(*λ*) is the net repulsive pressure and *λ* (dm) is the distance between two adjacent particles. In double-layer theory, *P*_*EDL*_(*λ*) can be calculated as per Eq. (4)^26,37^:

4 $$P_{EDL} (\lambda ) = 2RTf_{0} \left\{ {\cosh \left[ {\frac{ZF\varphi (\lambda /2)}{{RT}}} \right] - 1} \right\}$$

where, *R* (J mol^−1^ K^−1^), *F* (C mol^−1^) and *Z* represent the gas constant, Faraday’s constant and the cation valence, respectively. *φ*(*λ*/2) (V) is the potential at the middle point of overlapping EDLs for two adjacent particles. There is a mathematical relation between *φ*(*λ*/2) and surface potential that can be expressed as^10^:

5 $$\begin{aligned} & \frac{\pi }{2}\left[ {1 + \left( \frac{1}{2} \right)^{2} e^{{\frac{2ZF\varphi (\lambda /2)}{{RT}}}} + \left( \frac{3}{8} \right)^{2} e^{{\frac{4ZF\varphi (\lambda /2)}{{RT}}}} } \right] - \arcsin e^{{\frac{{ZF\varphi_{0} - ZF\varphi \left( {\frac{\lambda }{2}} \right)}}{2RT}}} \\ & \quad = \frac{1}{4}\lambda \kappa e^{{\frac{{ - ZF\varphi \left( {\frac{\lambda }{2}} \right)}}{2RT}}} \\ \end{aligned}$$

where, *κ* (dm^−1^) is the Debye-Hückel parameter and *κ* = (8*πF*^2^*f*_0_/*εRT*)^1/2^ and *ε* is the dielectric constant of water (8.9 × 10^–10^ C^2^ J^−1^ dm^−1^). The *φ*_0_ (mV) is the surface potential, and it can be calculated in 1:1-type electrolytes as per Eqs. (6) and (7)^26^:

6 $$\varphi_{0} = - \frac{2RT}{{ZF}}\ln \left( {\frac{1 - u}{{1 + u}}} \right)$$

7 $$\frac{\kappa \sigma }{{f_{0} }} = 1 + \frac{4}{1 + u} - \frac{4}{{1 + e^{ - 1} u}}$$

where, *σ* (C m^−2^) is the surface charge density and *u* = tanh(*ZFφ*_0_/*RT*) stands for a temporary parameter. The *φ*_0_ for 2:1-type electrolyte is shown in Eq. (8):^38:^

8 $$\sqrt {\frac{RT\varepsilon }{{2\pi }}\left[ {{\text{CCC}}\left( {a_{ + } e^{{ - \frac{{2F\varphi_{0} }}{RT}}} + 2a_{ - } e^{{ - \frac{{F\varphi_{0} }}{RT}}} - 1} \right)} \right]} = 0.965\frac{{{\text{CEC}}}}{S}$$

where, *a*_+_ and *a*_−_ are the cation and anion activities, respectively, and *S* (dm^2^ g^−1^) is the specific surface area. *CEC* is the cation exchange capacity.

To estimate long-range van der Waals force, Eq. (9) is the value used by the aid^39^.

9 $$P_{vdW} (\lambda ) = - \frac{A}{0.6\pi }(10\lambda )^{ - 3}$$

where, *A* (J) is the effective Hamaker constant. In this study, we use 6.99 × 10^–20^ J as the Hamaker constant for black soil colloid^25^. From this equation, the *P*_*vdW*_(*λ*) depends only on the inter-particle distance and the Hamaker constant.

In simulating the DLVO force of BSC aggregation in K^+^ system, the surface potential *φ*_0_ of colloid particles can be obtained by Eqs. (6) and (7). The potential value is then brought into Eq. (5) to compute the middle point potential *φ*(*λ*/2). Then, this *φ*(*λ*/2) value is used to compute the *P*_*EDL*_(*λ*) at different electrolyte concentrations through Eq. (4). Finally, the net DLVO force is obtained by summing the *P*_*EDL*_(*λ*) and *P*_*vdW*_(*λ*) obtained by Eq. (9). While in Mg^2+^ and Ca^2+^ electrolyte systems, the DLVO force calculation process is the same as K^+^ system, except that Eq. (8) is used to calculate *φ*_0_ instead of Eqs. (6) and (7).

Thus, the energy barrier Δ*W* (*kT*) for particle aggregation is expressed in Eq. (10):^16:^

10 $$\Delta W = \frac{s}{kT}\int\limits_{x}^{y} {P_{DLVO} (\lambda )d\lambda }$$

where, In the *P*(*λ*) ~ *λ* curve, the lower limit when *P*(*λ*) = 0 is defined as *x* (m), and the upper limit is defined as *y* (m). In addition, there is a potential barrier in the domain of *λ* = *x* to *y*. *s* (m^2^) represents the average area passing through the repelling space in this interval from *λ* = x to y.

## Results and discussion

### Aggregation kinetics of BSCs in monovalent and divalent electrolytes

**Figure 2:**
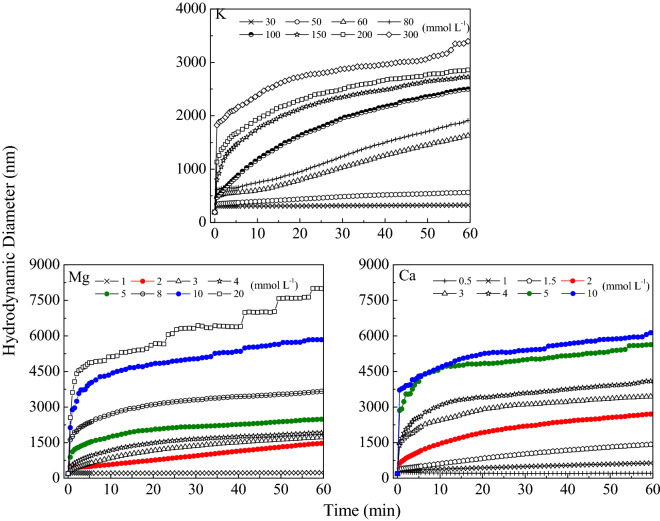
The average effective hydrodynamic diameters of the BSC particle aggregates changing with the experimental time.

The average hydrodynamic diameter of BSC aggregates in the presence of KCl, CaCl_2_ and MgCl_2_ were measured at a range of electrolyte concentrations to explore the electrolyte-dependent aggregation heterogeneity (Fig. 2). According to the effective collision probability, two aggregation mechanisms for colloidal particle aggregation can be summarized. One mechanism, corresponding to fast aggregation, is considered as diffusion limited cluster aggregation (DLCA). The other, corresponding to slow aggregation, is the reaction limited cluster aggregation (RLCA). In the RLCA mechanism, the aggregate diameter increases with time are linear dependence, while it increases exponentially with the increase in time in the DLCA mechanism^25,40–42^. The aggregation of BSCs increased with the increase in electrolyte concentration because the cations neutralized the negative surface charge of BSCs, thereby reducing the repulsive force between particles and enhancing aggregation. Divalent cations (Ca^2+^ and Mg^2+^) could induce a rapid and an intense aggregation of BSCs than monovalent cations (K^+^) even at far lower ionic concentrations. For example, the BSCs remained stable in K^+^ systems at an ionic concentration of 10 mmol L^−1^, while rapid aggregation occurred in Ca^2+^ and Mg^2+^ systems at the same ionic concentration; additionally, Ca^2+^ had a stronger effect in comparison to that of Mg^2+^. Compared with monovalent cations, a much lower concentration of divalent cations induced a drastic BSC aggregation, which may be because high-valent cations can make the surface charge of BSCs less negative through the increased charge screening effect, which benefits aggregation^43^. Previous studies have found that the stronger the surface charge, the more significant the shielding effect of high-valent cations^18^.

Contrary to the Schulze-Hardy rule, cations with the same valence can have a significantly different coagulation ability^1,44^. The Schulze-Hardy rule, emphasizing the theoretical coagulation value as determined by the valence of metal ions, implies that cations with the same valence have the same dispersion or coagulation ability. However, results of several experiment show that alkali-metal ions and alkaline earth-metal ions with the same valence have surprisingly different aggregation capabilities^26,27,34^. The data presented herein show that the BSC aggregation process was the strongest for Ca^2+^, followed by Mg^2+^, the lowest for K^+^. In summary, the dispersion stability of BSCs for the same electrolyte concentration were higher in KCl than that in MgCl_2_ and CaCl_2_ solutions. The effects of these cations on BSC aggregation are in the order Ca^2+^ > Mg^2+^ > K^+^, suggesting that there are strong specific ion effects in the BSC aggregation process.

### FESEM images and *d*_*f*_ of BSC aggregates with K^+^, Mg^2+^ and Ca^2+^ electrolytes

**Figure 3:**
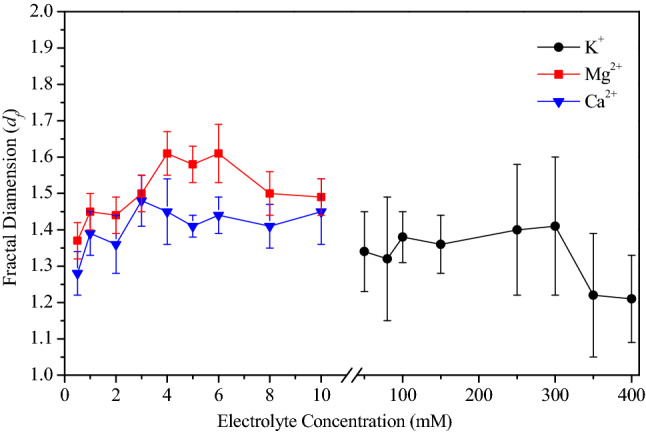
Fractal dimension of BSC aggregates in different K^+^, Mg^2+^ and Ca^2+^ electrolyte concentrations. Data are expressed as the mean ± standard deviation of triplicate measurements.

The different aggregation process not only affect the dispersion stability of soil colloids but also alter the structural characteristics and surface topography of aggregates. Thus, the *d*_*f*_ was measured to characterize the BSC aggregate structure. A relatively high *d*_*f*_ indicates that the aggregate structure is dense and the porosity is small; a low *d*_*f*_ indicates that the structure is loose, open and has a large porosity. The fractal dimension of aggregates under different concentrations of K^+^, Mg^2+^ and Ca^2+^ electrolytes were list in Fig. 3. Under extremely low electrolyte concentrations, the aggregation process is very weak. At this time, the aggregates size is small with insignificant fractal characteristics, and the fractal dimension is low. With the increase of electrolyte concentration, the aggregation process intensifies, and forming large aggregates with significant fractal characteristics. However, when the electrolyte concentration further increases, the repulsive barrier between particles disappears completely. At this time, the aggregation occurs immediately once the particles collide. Which is not conducive to form a regularly arranged fractal structure, so the fractal dimension of aggregates decreases. This result is consistent with the conclusion that RLCA contributes to the formation of aggregates with less porosity, compact structure and more prominent self-similar characteristics than DLCA^40,41^.

The data in Fig. 3 shows that the *d*_*f*_ of BSC aggregates in Mg^2+^ were all higher than that in Ca^2+^at the same ionic concentration. This trend suggests that ion-induced week coagulation is more likely to cause a compact aggregate structure. For black soil colloid aggregates, the reason for the dense soil structure is the dense aggregates, and a denser structure of Mg^2+^-aggregates means stronger repulsive forces among soil particles in soil solutions. These repulsions create opportunities for the particles to be bonded through multiple collisions, thus, finding more stable sites. Therefore, Ca^2+^-aggregates with lower fractal dimensions may correspond to a less stable soil structure. On the other hand, the fractal dimension of Ca^2+^-aggregates is low, which represents larger aggregate volume and stronger anti-sticking properties, viscosity resistance as the aggregates move in the aqueous medium of soil pores; therefore, aggregates with lower fractal dimension may travel slower through a soil pore^14^.

**Figure 4:**
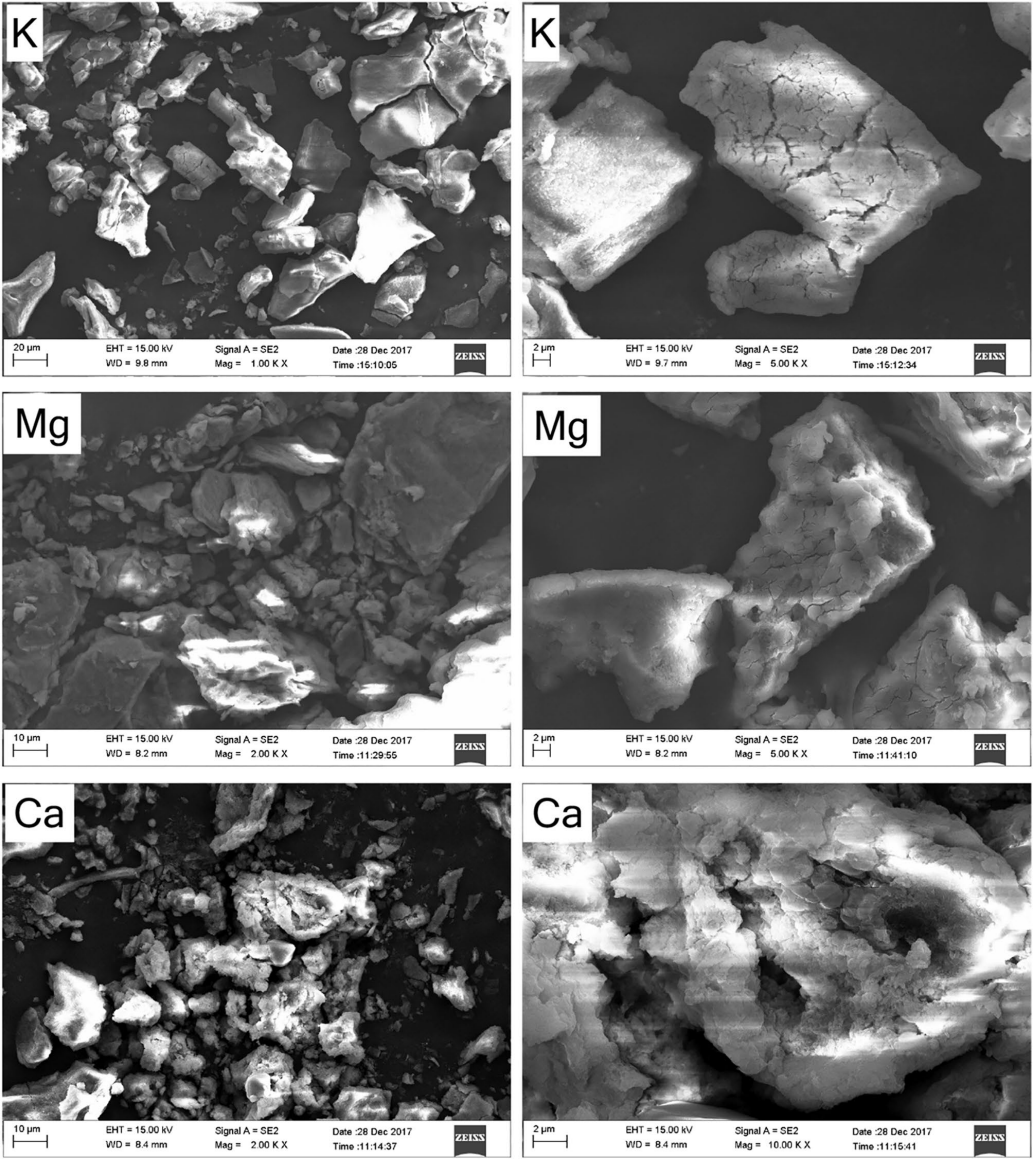
FESEM images of BSC aggregates with K^+^, Mg^2+^ and Ca^2+^ electrolytes.

The texture and shape of BSC aggregates, formed in different electrolyte solutions observed in FESEM and shown in Fig. 4, indicating that the aggregates are floc-like in Ca^2+^ solution, schistose-like in Mg^2+^ solution and block-like structure in K^+^ solutions, with small cracks and variable degrees of roughness. Furthermore, the BSC aggregates induced by K^+^ has a relatively smooth surface and clean edges. Comparatively, Mg^2+^ and Ca^2+^-induced aggregates take the shape of sheet or floc and have a rough edge, with small bumps and variable degrees of roughness. It is evident that the structural changes and enhanced aggregation of BSCs are primarily due to the cation-surface interactions in a strong electric field. Fractal dimension and FESEM imaging provided evidence for the presence of specific ion effects in BSC aggregate structure and morphology.

The fact that Ca^2+^ is more polarized than Mg^2+^ under a strong electric field of BSC, implies that Ca^2+^ rather than Mg^2+^ and K^+^ has a higher probability of appearing in proximity to the charged surface. Therefore, the shielding effect of the electric field is greater in the case of Ca^2+^ solution, resulting in a decrease in the activation energy of BSC particle aggregation. Thus, the ion-surface interaction in the electrolyte field should account for cations in the same valence that have significantly different effects on BSC dispersion stability, BSC aggregate structure and surface topography.

### CCCs of monovalent and divalent cations in BSC aggregation

**Figure 5:**
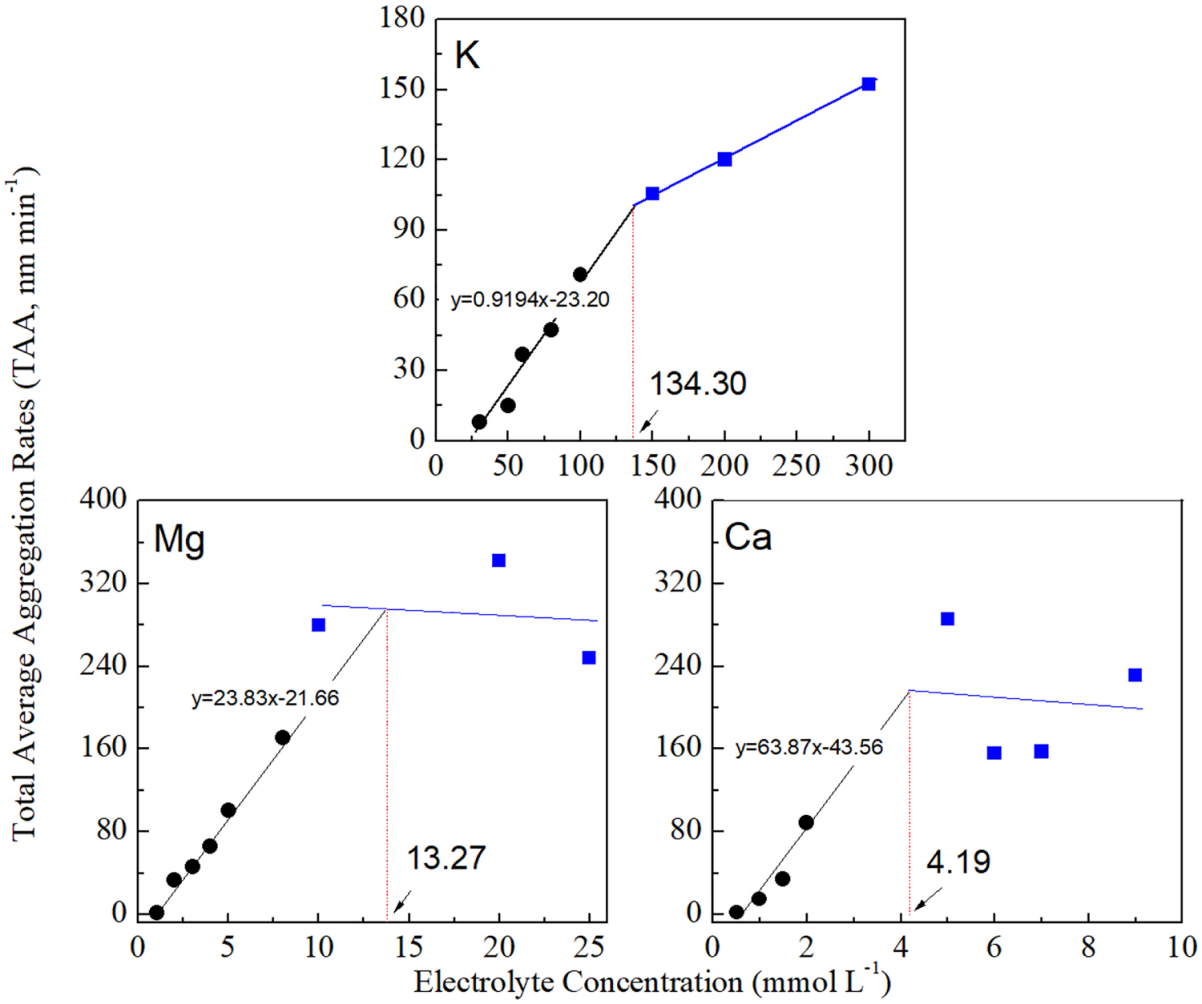
The TAA rates for the aggregation of the BSC particles as a function of the electrolyte concentration in K^+^, Mg^2+^ and Ca^2+^ electrolytes.

According to the DLS experiments, we made the data into a dynamic view during aggregation and disaggregation, which revealed the interactions and conduced to assessment of velocities of mineral assemblages. The data presented in Fig. 5 show that all of the TAA rates of BSCs in different electrolyte solutions increased significantly at the low concentration ranges but either plateaued or increased gradually at higher concentrations. In each case, the TAA rates of each electrolyte solution can be mathematically expressed by two linear functions both for the low and high concentrations, the two straight lines of the electrolyte concentration will intersect at the intersection point, and it corresponds to CCC. Where the TAA rate was nearly consistent or slightly changed above the CCC, in which the colloid aggregation was DLCA. The portion at which the TAA rate increased linearly with increase in time was RLCA, in which only partial colloid collision resulted in aggregation due to the energy barriers originating from the repulsive force.

The CCC values are found to be equal to 134.3, 13.3 and 4.2 mmol L^−1^ for KCl, MgCl_2_ and CaCl_2_, respectively. The CCC can quantify the ability of different cations to cause soil colloid aggregation. Through analysis of the data of CCCs at various electrolyte concentrations drew a conclusion that CCC changed along with the ionic valence and ion specificity. For coagulation, divalent ions was obviously superior to that of monovalent ions. However, for the same valence cations, CCC_Mg_ was 3.2 times as much as that for CCC_Ca_. Similar results have been reported in a previous study on mineral montmorillonite aggregation^34^. The CCCs of Ca^2+^ and Mg^2+^ were 2.38 and 7.99 mmol L^−1^, respectively, on montmorillonite aggregation, and CCC_Mg_ was nearly 3 times as much as that for CCC_Ca_, indicating that the CCC of these two cations in the natural soil particle aggregation process is larger than that in pure montmorillonite because of the presence of organic components in natural soil. The organic components cause a stronger electric field around the soil colloid particles. However, the difference between the natural soil and the mineral particles for CCC_Mg_ is always three times that for CCC_Ca_, implying that the coagulation ability of Ca^2+^ and Mg^2+^ is weakened to the same extent. Therefore, the difference in coagulation ability between Ca^2+^ and Mg^2+^ is not based on the composition of the particle system. Further, it is not clear that whether the difference was caused by the variations in particle size including ionic radius and ion hydration radius? Yet, this difference is contrary to the variation in ion volume reported in previous studies, and the fact that the hydrated radii of Mg^2+^ and Ca^2+^ were 0.30–0.47 nm and 0.41–0.42 nm, respectively^45–47^. Therefore, large differences in CCC for aggregation among this pair of cations did not result from the minute differences in volume because their hydrated radii are also similar. As what the TAA rates and CCC values had indicated, specific ion effects are apparent for different base cations (K^+^, Mg^2+^ and Ca^2+^). Therefore. For the purpose of in-depth understanding of the specific ion effects in the BSC aggregation process, it is thus evaluating the activation energies in KCl, MgCl_2_ and CaCl_2_ solutions that counts.

### The interaction activation energy between BSCs with monovalent and divalent electrolytes

Large differences in activation energy between BSCs are also observed in the aggregation kinetics induced by monovalent and divalent cations. In general, the higher the activation energy, the stronger is the soil colloidal dispersion stability. In various cationic solutions, Eq. (2) are used to theoretically calculate the activation energy of BSC particle aggregation. Specific equations for different solutions are as follows:

In KCl: Δ*E* (*f*_0_) (J mol^−1^) = − *kT* ln (0.0092*f*_0 _− 0.23),  *f*_0_ ≤ 134.30 mmol L^−1^.

In MgCl_2_: Δ*E*(*f*_0_) (J mol^−1^) = − *kT* ln (0.081*f*_0 _− 0.07),  *f*_0_ ≤ 13.27 mmol L^−1^.

In CaCl_2_: Δ*E*(*f*_0_) (J mol^−1^) = − *kT* ln(0.29*f*_0 _− 0.19),  *f*_0_ ≤ 4.19 mmol L^−1^.

**Figure 6:**
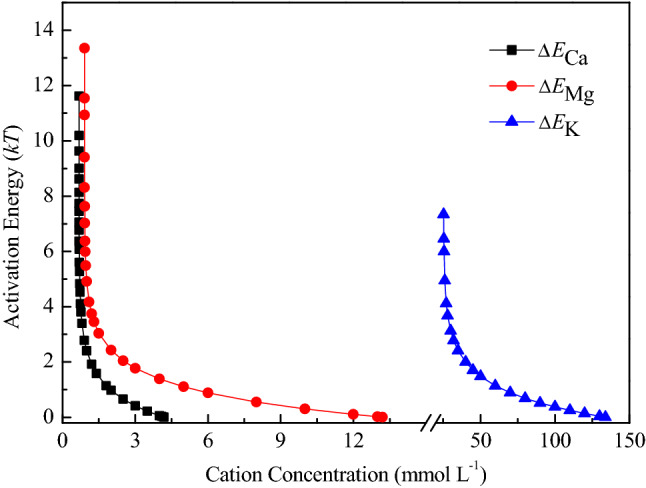
The activation energies ∆*E*(*f*_0_) for the aggregation of the black soil colloid particles in K^+^, Ca^2+^ and Mg^2+^ solutions.

The functions between the activation energy ∆*E*(*f*_0_) and cation concentration *f*_0_ given above are also plotted in Fig. 6. These data show that no matter what the value of the cation concentration (≤ CCC) is given, the activation energies ∆*E*(*f*_0_) of soil particles in these cation solutions are significantly different. The activation energy is 0.14 *kT* for 120 mmol L^−1^ K^+^. In an electrolyte solution with a concentration of 2 mmol L^−1^, the activation energies in Ca^2+^ and Mg^2+^ solutions are 0.98 and 2.43 *kT*, respectively, and the activation energy for particles in Mg^2+^ is 2.48 times as much as that for Ca^2+^. However, the change in difference to 24.56 times at an electrolyte concentration of 4 mmol L^−1^ suggests that cations with the same valence have a large difference in affecting soil colloid dispersion stability. Additionally, this difference varies greatly with cation concentration. Further, when the activation energy between soil colloids is 5 *kT* in each cation system, the required cation concentrations are 0.71 mmol L^−1^ Ca^2+^, 0.99 mmol L^−1^ Mg^2+^ and 25.96 mmol L^−1^ K^+^. Activation energy is a quantitative measure of the underlying specific ion effects change in the order of Ca^2+^ < Mg^2+^ < K^+^, and CCC values has a good consistency with the above results on the aggregation rates.

By analyzing the activation energies, we can understand intuitively the change trends of the aggregation rates and CCC values in different cation solutions. The K^+^ results show the lowest aggregation rate and the highest CCC value for the aggregation of BSC particles, and the reason for the results is that K^+^ produces the highest activation energies for this aggregation process. As the activation energies decrease from K^+^ to Mg^2+^ and finally to Ca^2+^, the corresponding aggregation rates show an increasing trend, whereas the CCC values show a decreasing trend.

**Figure 7:**
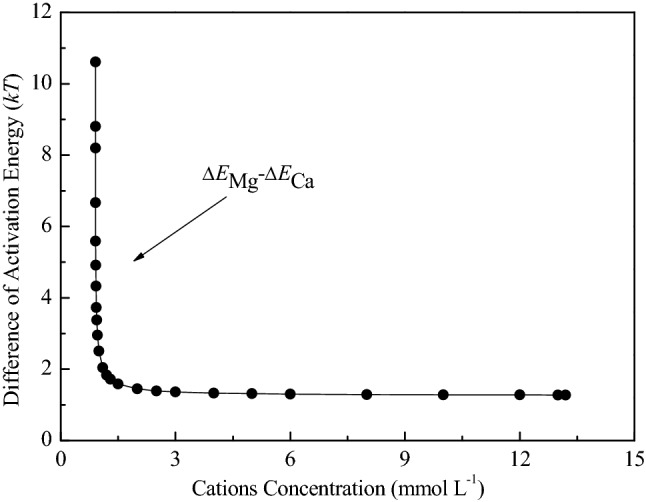
The correlations of the activation energy differences [∆*E*_*Mg* _− ∆*E*_*Ca*_] between cations Mg^2+^ and Ca^2+^ with the electrolyte concentration *f*_0_.

Further, the difference in the activation energy of particles between Mg^2+^ and Ca^2+^ in a certain electrolyte concentration is depicted in Fig. 7. It shows that for Mg^2+^ and Ca^2+^, their activation energy differences increase sharply with the decrease of ion concentrations. That is the decrease of ion concentration leads to an obvious increase of specific ion effects. Furthermore, a decrease in electrolyte concentration enhances the electric field near negatively-charged soil particle surfaces. Such a strong electric field enhances cation polarization, and is indicative of the significant role played by the polarization effect. The polarization, also observed in montmorillonite interactions and ion adsorption in previous studies^16,27,48^, indicates that for a real soil system, there is still a nonclassical polarization of cations directly related to the electric field near the soil colloidal surface. Therefore, the ionic polarization at the interface is a non-negligible non-DLVO factor.

### The influence of electrolyte concentration on the zeta potential of BSC aggregates

**Figure 8:**
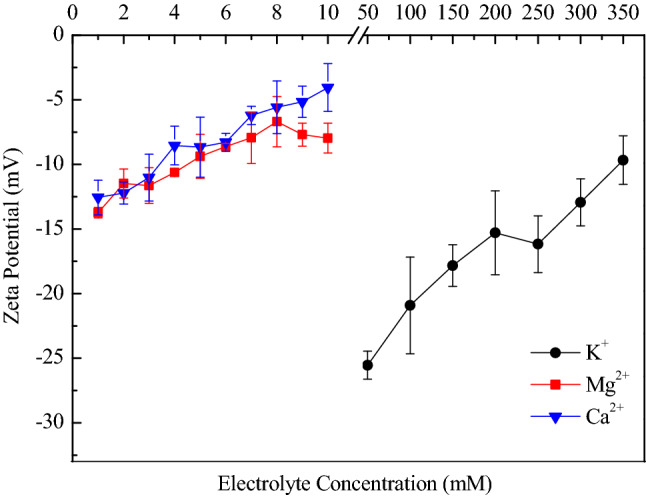
The zeta potential of BSC aggregates in different concentrations of K^+^, Mg^2+^ and Ca^2+^ electrolytes. Data are expressed as the mean ± standard deviation of triplicate measurements.

Change in the zeta potential may indirectly reflect the change in the surface charge density. The zeta potential of BSCs were all negative for KCl, MgCl_2_ and CaCl_2_ solutions within the electrolyte concentration ranges; furthermore, divalent cations in comparison to that of monovalent cations make the zeta potential of BSCs less negative (Fig. 8). Unlike monovalent ions of K^+^ influencing zeta potential primarily through the electric screening effect, divalent ions of Mg^2+^ and Ca^2+^ adsorb to the surface sites not only by the attractive electrostatic forces but also via the formation of surface complexes with functional groups^49^. However, the zeta potential of BSCs in Mg^2+^ was higher than that in Ca^2+^ for most electrolyte concentrations, although they have the same ionic valence. Further, the zeta potential is the shear plane potential of the charged colloid, and the distance of the shear plane from the surface in a monovalent cation solution is much greater than that in a divalent cation solution at the same zeta potential^50^. Several studies show that zeta potential is approximately 3.1–6.0 times lower than the surface potential (potential at the original plane of diffusion layer)^50^. The effect of ions on particle surface properties is more easily reflected on the surface potential rather than on the zeta potential.

The data in Fig. 8 shows that at relatively low electrolyte concentration (50 mmol L^−1^) of K^+^, zeta potential of BSCs is ~ − 27 mV. At this point, the BSC system is in a dispersed stable state, and the stability of the suspension is caused by the repulsive electrostatic forces between colloidal particles. With the increase of cation concentrations, the zeta potential of BSCs became less negative and causes a decreased repulsive force between the particles, indicating unstable suspensions, in agreement with the DLS test results. Colloidal particles, in suspensions with zeta potentials of more than + 20 mV or less than − 20 mV, are stable systems^51^. Some scholars define this threshold value at 25 mV and called it 25 mV rule^52^. Later, researchers estimat the CCCs using 25 mV as a threshold of stability, and they found that 25 mV rule is clearly unreliable for low CCCs (< 100 mV), especially for suspensions containing multivalent ions^18,53^. Here our current results further confirm its limitation.

### DLVO simulation of BSC aggregation in K^+^, Mg^2+^ and Ca^2+^ electrolytes

**Figure 9:**
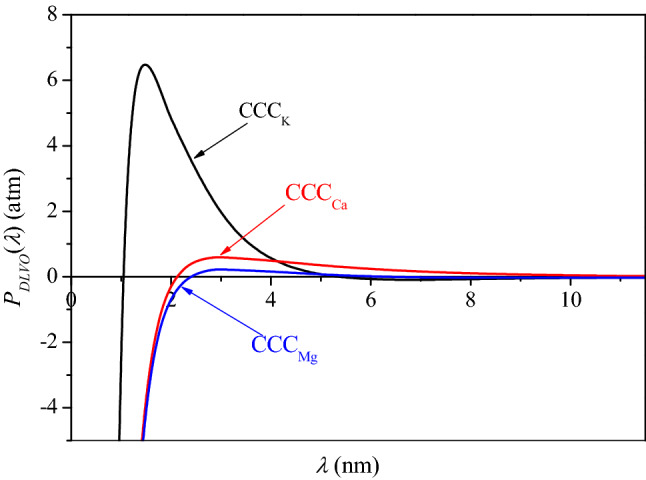
The classical *P*_*DLVO*_(*λ*) at CCCs of K^+^, Mg^2+^ and Ca^2+^, where curves in different colors represent the different cation systems; the areas of closed curves *P*_*DLVO*_(*λ*) versus *λ* were utilized to estimate the energy barrier, Δ*W* with a unit of *kT.*

The classic DLVO theory, can be used to reveal the stability of colloids, which is affected by van der Waals force and electrostatic force Waals and repulsive electrostatic interactions. In the present study, the CCC values of K^+^, Mg^2+^ and Ca^2+^ were obtained, and calculate the sum of DLVO pressure when *f*_0_ = CCC, and plot the result in Fig. 9. When *f*_0_ = CCC, the effective collision probability of the particles equals one; that is, aggregation will occur once the particles collide. Therefore, the repulsive barrier between the particles is zero at that time. However, as shown in Fig. 9, the energy barrier Δ*W* is obviously not zero at CCC_K_, CCC_Ca_ and CCC_Mg_ according to the classical DLVO theory. Meanwhile, there will be a certain deviation between the aggregation results and the classic DLVO simulation. This deviation value can verify that non-DLVO factors also play a key role in the aggregation process. While it may be difficult to accurately analyze what the non-DLVO factors are, the impact of non-DLVO factors has been reported by others^20^.

While considering the non-DLVO factors, it is also necessary to consider the additional energy in the ion-surface interaction (Eq. 11)

11 $$w_{i} (total) = Z_{i} F\varphi_{0} + w_{i} (add) = \gamma_{i} Z_{i} F\varphi_{0}$$

where *w*_*i*_ (total) is the total ion-surface interaction energy of the *i*th ion species, *Z*_*i*_*Fφ*_0_ is the classic electrostatic interaction energy, *w*_*i*_ (add) represents the additional interaction energy of nonclassical DLVO factors, and *γ*_*i*_ is the effective ionic charge coefficient of the *i*th ion species. Regardless of which forementioned effects cause the additional interaction energy of the cation to be changed, the contribution of the total ion-surface interaction energy *w*_*i*_ (total) can be measured by the change in the number of charges. Thus, the cationic apparent charge changes from *Z*_*i*_ into *γ*_*i*_*Z*_*i*_ in the modified calculation. In the aggregation process of a given charged particle, the van der Waals attraction *P*_*vdW*_ is a certain value, and the reliable estimation of the energy barrier Δ*W* relays on the correct electrostatic repulsion, *P*_*EDL*_, regardless of the cation. Within Eqs. (4)–(8), *Z*_*i*_ is replaced by *γ*_*i*_*Z*_*i*_ to describe the modified electrostatic repulsive interaction. The γi is a key parameter that can determine the strength of additional interaction energy of the *i*th ion species, and further determines the energy barrier for particle aggregation.

**Figure 10:**
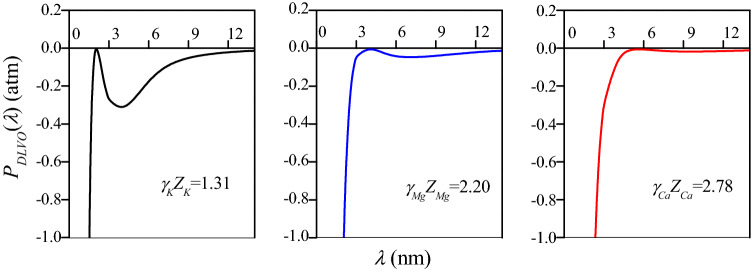
The modified *P*_*DLVO*_(*λ*) at CCCs of K^+^, Mg^2+^ and Ca^2+^, where curves in different colors represent the different cation systems; the areas of closed curves *P*_*DLVO*_(*λ*) versus *λ* were utilized to estimate the energy barrier and Δ*W* = 0 *kT* at *f*_0_ = CCC_K_ = 134.30, CCC_Mg_ = 13.27 and CCC_Ca_ = 4.19 mmol L^−1^, respectively.

With a series of assumed *γ*_*i*_ values, the modified net DLVO force pressure, *P*_*DLVO*_(*λ*), in CCC_K_, CCC_Ca_ and CCC_Mg_ are quantitatively calculated with a combination of Eqs. (3)–(10). Wherein, the coefficient makes the modified energy barrier equal to zero, Δ*W* = 0, is sought after from the *γ*_*K*_, *γ*_*Ca*_, and *γ*_*Mg*_ by taking the non-DLVO factors into account. The data in Fig. 10 show that the energy barrier of zero is derived using *γ*_*i*_ of 1.31, 1.10, and 1.39 for BSC aggregation in K^+^, Mg^2+^ and Ca^2+^ electrolytes, respectively, as non-DLVO factors are considered.

The charge numbers considered non-DLVO factors were in the order *γ*_*Ca*_*Z*_*Ca*_ (2.78) > *γ*_*Mg*_*Z*_*Mg*_ (2.20) > *γ*_*K*_*Z*_*K*_ (1.31) in natural BSC suspension. In addition, the same cation has different charge coefficients in different systems. In the pure mineral model system, the charge numbers of Ca^2+^ are 3.38 and 3.92 in montmorillonite and humic acid-montmorillonite complexes and that of Mg^2+^ is 2.38 in montmorillonite, respectively^16,34^. The difference of charge numbers between the model system and natural soil system may due to two reasons. Firstly, the colloid surface types are different. The surface of montmorillonite is dominated by permanent charges. While, the surface of natural black soil is more complex including both permanently and variably charged surface. Secondly, the composition of colloidal substances is different. BSC contains clay minerals such as montmorillonite and illite, as well as organic matter, metal oxides and other substances. Studies have shown that divalent ions have different affinities with the surface of humic acid, leading to different aggregation process^19^. Researchers proposed that there is a similar non-classical polarization of anions in the yellow soil colloid system^27^. They concluded that interaction of anions with cations and proton affinities in the electric field was the influencing factors for anion specificities. Therefore, it is necessary to carry out research on soil colloid aggregation with various properties. This means that the effects of non-DLVO factors vary in different systems. All the non-DLVO factors combine to dominate the colloid particle behavior. The effective ionic charge coefficient fitting based on DLS determination accurately quantifies the intensity of non-DLVO forces during natural soil particle aggregation. This calculation provides a basis for further quantitative analysis of non-DLVO factor effects in other processes. The effective charge number described above can be used to supplement the calculation of related parameters considering the non-DLVO effects in the same system.

## Conclusions

K^+^, Mg^2+^ and Ca^2+^ cations strongly affect the BSC dispersion stability. Ion-surface interactions in a strong electrolyte field is a primary mechanism of cation effects on soil colloid interactions, causing the difference in colloid interaction energy and further affecting soil colloid dispersion stability. The data presented support the conclusion that: (1) pronounced specific ion effects were observed in BSC aggregation follow the sequence of Ca^2+^ > Mg^2+^ > K^+^. This sequence is in accord with the activation energy and zeta potential for BSC aggregation in different electrolytes; (2) the effective ionic charge numbers 1.31, 2.20, and 2.78 obtained from the ion specific particles’ aggregation kinetics in the presence of K^+^, Mg^2+^ and Ca^2+^ can be used to quantify all the non-DLVO factors effect on natural soil colloid aggregation; (3) the specific ion effects of cations further lead to the difference in the structure of the aggregates by affecting the aggregation process, and final affecting the morphology of the aggregates. The new background mechanism above strengthen the understanding of the processes affecting improvement of the stability of black soil aggregates.

## References

[1] Sumner ME (1999). Handbook of Soil Science.

[2] Yin XQ, Gao B, Ma LQ, Saha UK, Sun H, Wang GD (2010). Colloid-facilitated Pb transport in two shooting-range soils in Florida. J Hazard Mater..

[3] Henderson R, Kabengi N, Mantripragada N, Cabrera M, Hassan S, Thompson A (2012). Anoxia-induced release of colloid-and nanoparticle-bound phosphorus in grassland soils. Environ. Sci. Technol..

[4] Van den Bogaert R, Labille J, Cornu S (2015). Aggregation and dispersion behavior in the 0-to 2-µm fraction of Luvisols. Soil Sci. Soc. Am. J..

[5] Xia B, Qiu H, Knorr KH, Blodau C, Qiu RL (2018). Occurrence and fate of colloids and colloid-associated metals in a mining-impacted agricultural soil upon prolonged flooding. J. Hazard Mater..

[6] Brubaker SC, Holzhey CS, Brasher BR (1992). Estimating the water-dispersible clay content of soils. Soil Sci. Soc. Am. J..

[7] Wei XY, Pan DQ, Xu Z, Xian DF, Li XL, Tan ZY, Liu CL, Wu WS (2021). Colloidal stability and correlated migration of illite in the aquatic environment: The roles of pH, temperature, multiple cations and humic acid. Sci. Total Environ..

[8] Shainberg I, Levy GJ, Rengasamy P, Frenkel H (1992). Aggregate stability and seal formation as affected by drops' impact energy and soil amendments. Soil Sci..

[9] Seta AK, Karathanasis AD (1996). Water dispersible colloids and factors influencing their dispersibility from soil aggregates. Geoderma.

[10] Hu FN, Liu JF, Xu CY, Wang ZL, Liu G, Li H, Zhao SW (2018). Soil internal forces initiate aggregate breakdown and splash erosion. Geoderma.

[11] Hu FN, Li S, Xu CY, Gao XD, Miao SH, Ding WQ, Liu XM, Li H (2019). Effect of soil particle interaction forces in a clay-rich soil on aggregate breakdown and particle aggregation. Eur. J. Soil Sci..

[12] Aurell CA, Wistrom AO (2000). Coagulation of kaolinite colloids in high carbonate strength water. Colloid Surface..

[13] Petosa AR, Jaisi DP, Quevedo IR, Elimelech M, Tufenkji N (2010). Aggregation and deposition of engineered nanomaterials in aquatic environments: Role of physicochemical interactions. Environ. Sci. Technol..

[14] Tian R, Li H, Zhu HL, Liu XM, Gao XD (2013). Ca^2+^ and Cu^2+^ induced aggregation of variably charged soil particles: A comparative study. Soil Sci. Soc. Am. J..

[15] Chen KL, Mylon SE, Elimelech M (2006). Aggregation kinetics of alginate-coated hematite nanoparticles in monovalent and divalent electrolytes. Environ. Sci. Technol..

[16] Gao XD, Li H, Tian R, Liu XM, Zhu HL (2014). Quantitative characterization of specific ion effects using an effective charge number based on the gouy-chapman model. Acta Phys.-Chim. Sin..

[17] Katana B, Takács D, Szerlauth A, Sáringer S, Varga G, Jamnik A, Bobbink F, Dyson P, Szilagyi I (2021). Aggregation of halloysite nanotubes in the presence of multivalent ions and ionic liquids. Langmuir.

[18] Hegedűs T, Takács D, Vásárhelyi L, Szilágyi I, Kónya Z (2021). Specific ion effects on aggregation and charging properties of boron nitride nanospheres. Langmuir.

[19] Tiraferri A, Hernandez LAS, Bianco C, Tosco T, Sethi R (2017). Colloidal behavior of goethite nanoparticles modified with humic acid and implications for aquifer reclamation. J. Nanopart. Res..

[20] Grasso D, Subramaniam K, Butkus M, Strevett K, Bergendahl J (2002). A review of non-DLVO interactions in environmental colloidal systems. Environ. Sci. BioTechnol..

[21] Chorom M, Rengasamy P (1995). Dispersion and zeta potential of pure clays as related to net particle charge under varying pH, electrolyte concentration and cation type. Eur. J. Soil Sci..

[22] Grabbe A, Horn RG (1993). Double-layer and hydration forces measured between silica sheets subjected to various surface treatments. J. Colloid Interface Sci..

[23] Li H, Hou J, Liu XM, Li R, Zhu HL, Wu LS (2011). Combined determination of specific surface area and surface charge properties of charged particles from a single experiment. Soil Sci. Soc. Am. J..

[24] Chen KL, Mylon SE, Carstens JF, Bachmann J, Neuweiler I (2019). A new approach to determine the relative importance of DLVO and non-DLVO colloid retention mechanisms in porous media. Colloid Surface A..

[25] Zhu X, Chen H, Li W, He Y, Brookes PC, Xu J (2014). Aggregation kinetics of natural soil nanoparticles in different electrolytes. Eur. J. Soil Sci..

[26] Luo YX, Gao XD, Tian R, Li H (2018). Approach to estimation of hamaker constant as taking hofmeister effects into account. J. Phys. Chem. C..

[27] Tian R, Yang G, Li H, Gao XD, Liu XM, Zhu HL, Tang Y (2014). Activation energies of colloidal particle aggregation: Towards a quantitative characterization of specific ion effects. Phys. Chem. Chem. Phys..

[28] Dong HR, Lo IMC (2013). Influence of humic acid on the colloidal stability of surface-modified nano zero-valent iron. Water Res..

[29] Tang Z, Cheng T (2018). Stability and aggregation of nanoscale titanium dioxide particle (nTiO_2_): Effect of cation valence, humic acid, and clay colloids. Chemosphere.

[30] Tisdall JM, Oades JM (1982). Organic matter and water-stable aggregates in soils. J. Soil Sci..

[31] Sevink J, Verstraten JM, Jongejans J (1998). The relevance of humus forms for land degradation in Mediterranean mountainous areas. Geomorphology.

[32] Bolt GH (1955). Ion adsorption by clays. Soil Sci..

[33] Liu XM, Li H, Du W, Tian R, Li R, Jiang XJ (2013). Hofmeister effects on cation exchange equilibrium: Quantification of ion exchange selectivity. J. Phys. Chem. C..

[34] Gao XD, Tian R, Liu XM, Zhu HL, Tang Y, Xu CY, Shah GM, Li H (2019). Specific ion effects of Cu^2+^, Ca^2+^ and Mg^2+^ on montmorillonite aggregation. Appl. Clay Sci..

[35] Li KG, Zhang W, Huang Y, Chen YS (2011). Aggregation kinetics of CeO_2_ nanoparticles in KCl and CaCl_2_ solutions: Measurements and modeling. J. Nanopart. Res..

[36] Jia MY, Li H, Zhu HL, Tian R, Gao XD (2013). An approach for the critical coagulation concentration estimation of polydisperse colloidal suspensions of soil and humus. J. Soil Sediment..

[37] Li S, Li H, Xu CY, Huang XR, Xie DT, Ni JP (2013). Particle interaction forces induce soil particle transport during rainfall. Soil Sci. Soc. Am. J..

[38] Sposito G (1984). The Surface Chemistry of Soils.

[39] Li H, Peng XH, Wu LS, Jia MY, Zhu HL (2009). Surface potential dependence of the hamaker constant. J. Phys. Chem. C..

[40] Lin MY, Lindsay HM, Weitz DA, Ball RC, Klein R, Meakin P (1990). Universal reaction-limited colloid aggregation. Phys. Rev. A..

[41] Lin MY, Lindsay HM, Weitz DA, Klein R, Ball RC, Meakin P (1990). Universal diffusion-limited colloid aggregation. J. Phys. Condens. Matter..

[42] González AE (1993). Universality of colloid aggregation in the reaction limit: The computer simulations. Phys. Rev. Lett..

[43] Yang KJ, Chen BL, Zhu XY, Xing BS (2016). Aggregation, adsorption, and morphological transformation of graphene oxide in aqueous solutions containing different metal cations. Environ. Sci. Technol..

[44] Norrfors KK, Bouby M, Heck S, Finck N, Marsac R, Schäfer T, Geckeis H, Wold S (2015). Montmorillonite colloids: I. Characterization and stability of dispersions with different size fractions. Appl. Clay Sci..

[45] David F, Vokhmin V, Ionova G (2001). Water characteristics depend on the ionic environment. Thermodynamics and modelisation of the aquo ions. J. Mol Liq..

[46] Kiriukhin MY, Collins KD (2002). Dynamic hydration numbers for biologically important ions. Biophys. Chem..

[47] Tansel B (2012). Significance of thermodynamic and physical characteristics on permeation of ions during membrane separation: Hydrated radius, hydration free energy and viscous effects. Sep. Purif. Technol..

[48] Xu CY, Li H, Hu FN, Li S, Liu XM, Li Y (2015). Non-classical polarization of cations increases the stability of clay aggregates: Specific ion effects on the stability of aggregates. Eur. J. Soil Sci..

[49] Ludwig C, Schindler PW (1995). Surface complexation on TiO_2_: I. Adsorption of H+ and Cu^2+^ ions onto TiO_2_ (Anatase). J. Colloid Interf. Sci..

[50] Ding WQ, Liu XM, Song L, Li Q, Zhu QH, Zhu HL, Hu FN, Luo YX, Zhu LH, Li H (2015). An approach to estimate the position of the shear plane for colloidal particles in an electrophoresis experiment. Surf. Sci..

[51] Duman O, Tunç S (2009). Electrokinetic and rheological properties of Na-bentonite in some electrolyte solutions. Micropor. Mesopor. Mat..

[52] Vallar S, Houivet D, El Fallah J, Kervadec D, Haussonne JM (1999). Oxide slurries stability and powders dispersion: Optimization with zeta potential and rheological measurements. J. Eur. Ceram. Soc..

[53] Galli M, Sáringer S, Szilágyi I, Trefalt G (2020). A simple method to determine critical coagulation concentration from electrophoretic mobility. Colloid Interface..

